# C-type natriuretic peptide regulates endochondral bone growth through p38 MAP kinase-dependent and – independent pathways

**DOI:** 10.1186/1471-213X-7-18

**Published:** 2007-03-20

**Authors:** Hanga Agoston, Sameena Khan, Claudine G James, J Ryan Gillespie, Rosa Serra, Lee-Anne Stanton, Frank Beier

**Affiliations:** 1 CIHR Group in Skeletal Development and Remodeling, Department of Physiology and Pharmacology, University of Western Ontario, London, ON, N6A 5C1, Canada; 2 Department of Cell Biology, University of Alabama, Birmingham, USA

## Abstract

**Background:**

C-type natriuretic peptide (CNP) has recently been identified as an important anabolic regulator of endochondral bone growth, but the molecular mechanisms mediating its effects are not completely understood.

**Results:**

We demonstrate in a tibia organ culture system that pharmacological inhibition of p38 blocks the anabolic effects of CNP. We further show that CNP stimulates endochondral bone growth largely through expansion of the hypertrophic zone of the growth plate, while delaying mineralization. Both effects are reversed by p38 inhibition. We also performed Affymetrix microarray analyses on micro-dissected tibiae to identify CNP target genes. These studies confirmed that hypertrophic chondrocytes are the main targets of CNP signaling in the growth plate, since many more genes were regulated by CNP in this zone than in the others. While CNP receptors are expressed at similar levels in all three zones, cGMP-dependent kinases I and II, important transducers of CNP signaling, are expressed at much higher levels in hypertrophic cells than in other areas of the tibia, providing a potential explanation for the spatial distribution of CNP effects. In addition, our data show that CNP induces the expression of NPR3, a decoy receptor for natriuretic peptides, suggesting the existence of a feedback loop to limit CNP signaling. Finally, detailed analyses of our microarray data showed that CNP regulates numerous genes involved in BMP signaling and cell adhesion.

**Conclusion:**

Our data identify novel target genes of CNP and demonstrate that the p38 pathway is a novel, essential mediator of CNP effects on endochondral bone growth, with potential implications for understanding and treatment of numerous skeletal diseases.

## Background

Bone formation occurs through the related, but distinct processes of intramembranous and endochondral ossification [1,2]. While the former is responsible for the formation of bones directly from precursor cells, such as the majority of the skull, the latter is responsible for the development of long bones, ribs, and vertebrae through a cartilage intermediate. In endochondral ossification, mesenchymal cells condense and begin to differentiate into chondrocytes, some of which later form the growth plate that controls longitudinal growth of endochondral bones [3]. The growth plate zones consists of resting, proliferating, and terminally differentiated hypertrophic chondrocytes, each of which are characterized by the expression of specific markers [4,5]. This organization of the growth plate and the coordinated proliferation and hypertrophy of chondrocytes are responsible for elongation of bones and eventually determine final bone length. Hypertrophic chondrocytes are thought to undergo apoptosis, and simultaneously their surrounding cartilaginous matrix is degraded and replaced by bony tissue, produced by cells entering through vascularization of hypertrophic cartilage. The intricate control mechanisms regulating the proliferation, differentiation and apoptosis of chondrocytes as well as the subsequent vascular invasion are not completely understood. However, disturbances of these processes can result in numerous diseases such as chondrodysplasias and other growth disorders, demonstrating the need for a better understanding of the pathways involved [4,6-9].

C-type natriuretic peptide (CNP) has recently been shown to be an important regulator of endochondral ossification. The dominant phenotype of CNP-deficient mice is dwarfism, as demonstrated by shortened long bones primarily due to reduced heights of proliferating and hypertrophic zones of the growth plate [10,11]. CNP also increases growth in mouse bone organ cultures [12,13]. More recently, loss-of-function mutations in *NPR2*, the gene encoding the CNP receptor, have been identified as cause of acromesomelic dysplasia type Maroteaux, an autosomal recessive chondrodysplasia in humans [14]. This CNP receptor is also known as guanylyl cyclase-B (GC-B) or NPR-B. Binding of CNP to GC-B results in increased intracellular cGMP, which can further activate downstream factors, such as cGKI and cGKII (cGMP-dependent protein kinase I and II) as well as phosphodiesterases (PDEs) that break down cGMP and camp and specific ion channels [15-18]. In addition, CNP can also bind to a different receptor, NPR-3 (natriuretic peptide receptor 3) that is thought to act as a decoy/clearance receptor serving to limit the effects of natriuretic peptides. Interestingly, mice deficient for cGKII show a similar, although not identical phenotype to CNP-deficient mice [19], and cGKII has been shown to be required for the effects of CNP overexpression in transgenic mice [20]. These data clearly identify cGKII as an essential mediator of CNP effects, but the signaling pathways downstream of cGKII, potential parallel pathways and the target genes conferring cartilage responses to CNP are not completely understood. However, recent studies showed that overexpression of CNP results in inhibition of the MEK1/2-ERK1/2 MAP kinase pathway and rescues the effects of an activating fibroblast growth factor receptor 3 mutation on endochondral bone growth [21-23].

MAP kinases are central signaling molecules in most eukaryotic cells that integrate extracellular signals leading to altered cell proliferation, differentiation, and transcription in many cell types, including chondrocytes [24,25]. For example, both the ERK and the p38 MAPK families have been shown to play important roles in controlling chondrocyte differentiation *in vitro *and *in vivo *[3,26,27]. In the current study we demonstrate, for the first time, an essential role for the p38 MAP kinase pathway in CNP signaling in cartilage and identify target genes of CNP in chondrocytes using genome-wide microarrays.

## Results

### CNP signaling enhances endochondral bone growth

**Figure 1:**
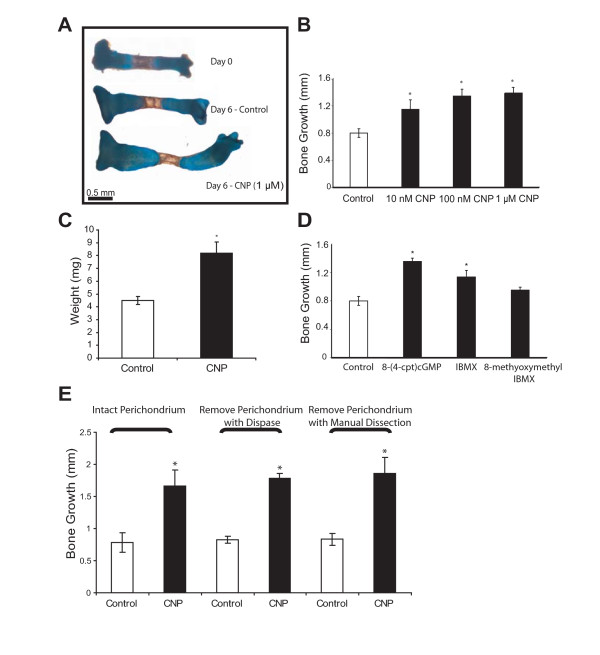
**CNP enhances endochondral bone growth**. Mouse E15.5 tibiae were harvested and cultured for six days in the presence of vehicle, CNP at the indicated concentrations, membrane-permeable 8-(4-cpt) cGMP (0.1 mM), the non-specific PDE inhibitor IBMX (0.1 mM), or a selective inhibitor of PDE I, 8-methooxymethyl, IBMX (10 μM). After six days in culture, vehicle and CNP-treated (1 μM) bones were stained with Alcian Blue and Alizarin Red and representative images are shown, in comparison to a freshly isolated tibia (**A**). Growth of tibiae over the culture period at indicated concentrations of CNP and treatments was measured (**B, D**), and the weight of bones was determined (**C**). CNP, 8-(4-cpt) cGMP and IBMX stimulated tibia growth, when compared to control conditions. E15.5 tibiae were isolated under three different conditions: perichondrium was left intact with very loose dissection, perichondrium was removed with dispase, and perichondrium was removed mechanically (**E**). Bones were then incubated with or without CNP (1 μM) for six days and bone growth was determined as change in bone length relative to day 1. Removal of the perichondrium did not influence the stimulatory effect of CNP on bone growth. All data represent means ± SD of three or four independent trials (p < 0.05).

We used an organ culture system of embryonic day 15.5 (E15.5) mouse tibiae to examine the effects of CNP on endochondral bone growth. Tibiae were cultured for six days in the presence of BSA (control) or different concentrations of CNP. 10^-8^, 10^-7 ^and 10^-6^M concentrations of CNP caused a 31%, 40%, and 42% increase, respectively, in longitudinal growth of tibiae (Fig. 1A,B). Treatment with 1 μM CNP almost doubled tibia weight relative to controls (Fig. 1C). Incubation of tibiae with 10^-4 ^M (8-(4-chlorophenylthio) cGMP stimulated tibia growth in a similar or stronger manner (55%) as CNP (Fig. 1D). A general inhibitor of phosphodiesterases (PDEs), 3-Isobutyl-1-methylxanthine (IBMX) at 10^-4 ^M, was used to study the role of PDEs in bone growth in the organ cultures. PDE inhibition stimulated longitudinal growth by 30% when compared to the control. In contrast, specific inhibition of PDE 1 by 8-methoxymethyl IBMX did not alter bone growth significantly, indicating that this enzyme is either not involved in regulating bone growth or can be functionally replaced by other proteins. These data demonstrate that CNP/cGMP signaling stimulates endochondral bone growth, while PDEs inhibit this process. Removal of the perichondrium by enzymatic digestion and/or manual dissection did not alter the response to CNP, demonstrating that the anabolic effects of CNP are independent of the perichondrium (Fig. 1E).

**Figure 2:**
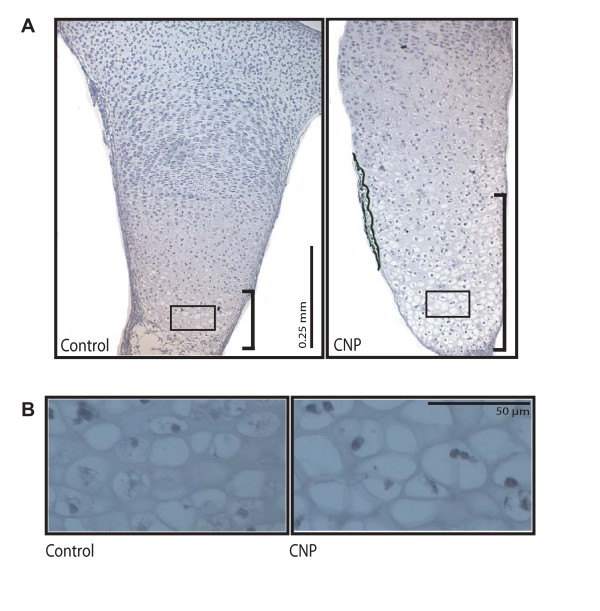
**CNP induces expansion of the hypertrophic zone**. Hematoxylin and Eosin staining of tibia sections after six days of culture with or without CNP (1 μM) showed differences in growth plate architecture, primarily in the hypertrophic zone. CNP treatment results in a vastly expanded hypertrophic zone (**A**; hypertrophic zones indicated by brackets). Magnification of cells in the hypertrophic zone (boxes from A) shows that individual chondrocytes are larger in CNP-treated tibiae (**B**).

At the histological level, the most significant effect of CNP was a marked expansion of the hypertrophic zone (Fig. 2A). This enlargement of the hypertrophic zone was accomplished by increases in both the number and maximal size of hypertrophic chondrocytes (Fig. 2B), in agreement with earlier studies [23].

### CNP-induced endochondral bone growth requires p38 MAP kinase signaling

**Figure 3:**
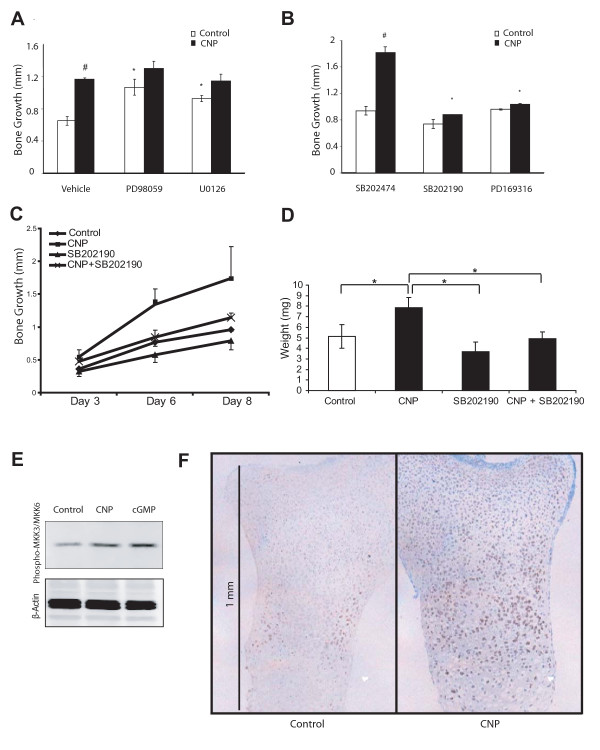
**Inhibition of the MEK1/2-ERK1/2 pathway stimulates tibia growth, while p38 MAPK is required for CNP-induced bone growth**. Mouse E15.5 tibiae were harvested and cultured for six days in the presence of control or CNP (1 μM) and vehicle (DMSO) or MEK1/2-ERK1/2 pathway inhibitors PD98059 (10 μM) and U0126 (10 μM) (**A**). Though both PD98059 and U0126 stimulated basal bone growth, inhibition of the MEK1/2-ERK1/2 pathway did not further enhance CNP-induced bone growth (*: p < 0.05 when comparing control/inhibitors to control/vehicle; #: p < 0.05 when comparing CNP/vehicle to control/vehicle; p > 0.05 when comparing CNP/vehicle to CNP/inhibitors). Tibiae were incubated with control or CNP and pharmacological inhibitors of the p38 MAPK pathway (SB202190 or PD169316, 10 μM each) or an inactive analog (SB202474, 10 μM) (**B**). p38 inhibition did not effect basal bone growth significantly, but did suppress CNP-induced bone growth (*: p < 0.05 when comparing CNP/inhibitors to CNP/SB202474; #: p < 0.05 when comparing CNP/SB202474 to control/SB202474). Bone growth was measured over an extended time course of eight days, showing that CNP continued to significantly influence growth on day 8, while SB202190 reversed these effects (**C**). Bones from each treatment were weighed under different conditions, and it was found that p38 inhibition reversed the effects of CNP on weight (**D**). Protein extracts from primary chondrocytes cultured with control, CNP (10^-6^M), or 8-(4-cpt) cGMP (0.1 mM) for 10 minutes were examined for phosphorylation of the p38 activators MKK3/6 by western blot analysis (**E**). Both treatments increased phosphorylation of MKK3/6, supporting the stimulation of p38 MAP kinase activity by CNP signaling. Immunohistochemistry with an antibody against phosphorylated p38 demonstrates markedly higher signal in CNP-treated tibiae when compared to control bones (**F**).

MAP kinases play multiple roles in chondrocyte differentiation and cartilage development [3]. We therefore examined a potential role of the MEK (MAP/ERK kinase) 1/2-ERK1/2 and p38 cascades in CNP-induced endochondral bone growth. In the absence of exogenous CNP, the pharmacological MEK1/2 inhibitors PD98059 and U0126 (10 μM each) stimulated tibia growth by 39% and 30%, respectively (Fig. 3A). While simultaneous addition of MEK inhibitors and CNP had maximal effects on bone growth, these effects were not statistically different from treatment with CNP alone. These data suggest that CNP and MEK1/2 act through a common pathway and are in agreement with recent studies demonstrating an inhibitory role of the MEK/ERK cascade in endochondral bone growth [26] and down-regulation of MEK/ERK activity by CNP [21-23].

We next examined whether p38 is involved in the effects of CNP on cartilage growth. Inhibition of p38 activity by two different compounds, PD169316 or SB202190 (10 μM each), did not affect basal endochondral bone growth, when compared to the inactive control compound SB202474 (10 μM) (Fig. 3B). In contrast, inhibition of p38 by SB202190 or PD169316 blocked CNP-induced growth (Fig. 3B). This effect was obvious by day 6 of culture and maintained by day 8 (Fig. 3C). Moreover, p38 inhibition completely reversed CNP effects on tibia weight (Fig. 3D), further demonstrating a requirement for p38 activity in CNP-induced endochondral bone growth. Next we examined whether CNP regulates the p38 pathway by investigating the phosphorylation of the kinases MKK3 (MAP kinase kinase3) and MKK6, direct and specific activators of p38. Western blotting with phospho-specific antibodies revealed that CNP and cGMP increase the phosphorylation of MKK3/6 in primary chondrocytes after 10 minutes of incubation (Fig. 3E), demonstrating that CNP signaling activates the p38 pathway in chondrocytes. Immunohistochemistry for phosphorylated (active) p38 showed little staining under control conditions, but demosntrated a strong increase in p38 phosphorylation in CNP-treated tibiae (Fig. 3F).

### CNP delays tibia mineralization in a p38-dependent manner

**Figure 4:**
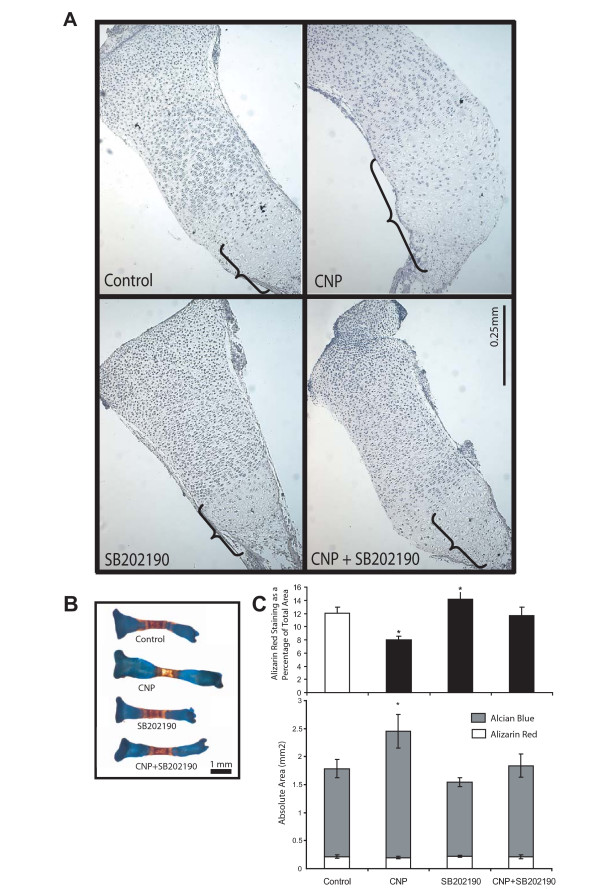
**p38 MAPK activity is required for CNP-induced hypertrophy**. E15.5 tibiae were isolated and incubated with or without CNP (1 μM) and DMSO or SB202190 (10 μM). Hematoxylin and Eosin staining of tibia sections after six days of culture show that p38 inhibition reversed CNP-induced expansion of the hypertrophic zone (**A**). Tibiae were stained with Alizarin Red and Alcian Blue, and representative images demonstrate increased bone growth by CNP and the reversal of these effects upon p38 inhibition (**B**). The area of the mineralized zone (red) was measured as absolute area (**C**, bottom) and as a percentage of total area (**C**, top), demonstrating that CNP-treated bones displayed significantly smaller mineralized area in relation to the whole bone area. This was reversed upon p38 inhibition. Representative images are shown, while all data represent means ± SD of four independent trials, each with six bones (p < 0.05).

To examine the effects of p38 inhibition on growth plate organization, we performed histological analyses of organ culture sections. As above, CNP stimulation caused an expansion of the hypertrophic zone of the growth plate, while SB202190 by itself did not have any marked effects (Fig. 4A). However, p38 inhibition suppressed the enlargement of the hypertrophic zone in response to CNP, providing further evidence for a requirement for p38 activity for the anabolic effects of CNP.

During dissections and analyses of organ cultures, we also noticed that CNP-treated bones were more fragile and appeared less mineralized. Alcian Blue/Alizarin Red staining of tibiae confirmed that the mineralized area was smaller in CNP-treated bones and displayed weaker Alizarin Red staining (Fig. 4B). We quantified the area of the mineralized (red) and cartilaginous (blue) regions of tibiae using digital image analyses. CNP treatment did increase the Alcian Blue-stained area considerably, without effects on the Alizarin Red-stained area (Fig. 4C). This resulted in a reduction of the mineralized area relative to the total area of the bone by about 30%. These data suggests that CNP-induced growth of cartilage is not matched by a corresponding expansion of the mineralized area and that CNP treatment delays the remodeling of hypertrophic cartilage. Inhibition of p38 activity by SB202190 resulted in a slight, but significant increase of the mineralized area (relative to total area) and reversed the effects of CNP on the Alcian Blue-stained area completely (Fig. 4C).

### Microarray analyses identify hypertrophic chondrocytes as main targets of CNP signaling

**Figure 5:**
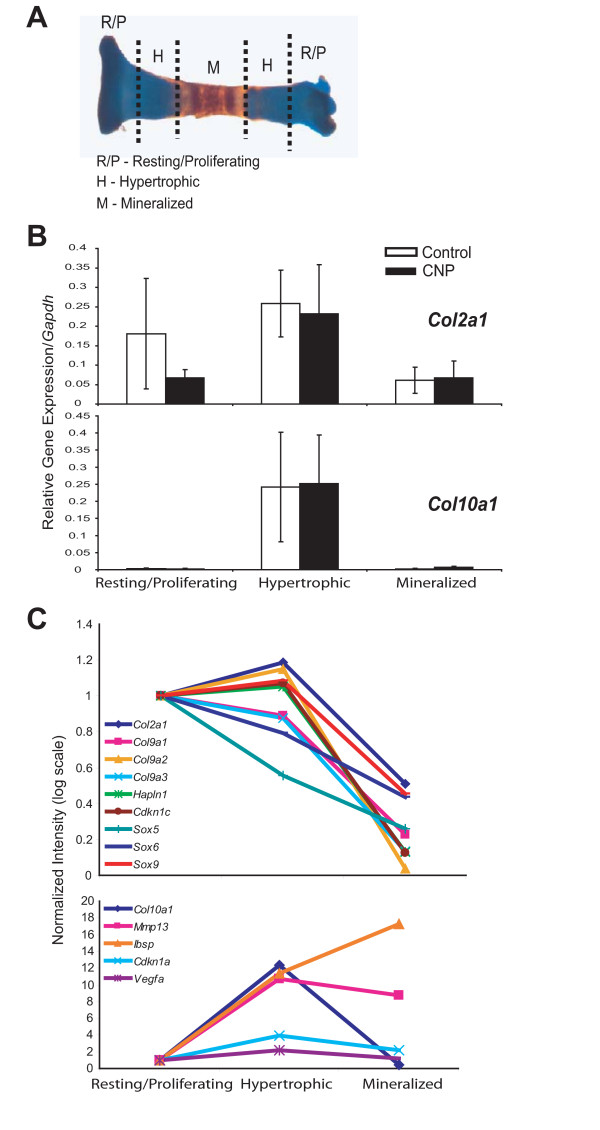
**Micro-dissection efficiently separates different growth plate zones from cultured tibiae**. E15.5 tibiae that were harvested and incubated with or without CNP (1 μM) for six days were micro-dissected into the resting/proliferating, hypertrophic, and mineralized regions as shown (**A**). Zones from approximately 24 bones were pooled together. RNA was isolated directly from micro-dissected tibia and analyzed by microarray as described in Materials and Methods. Real-time PCR analyses confirmed expected expression patterns of the cartilage markers *Col2a1 *and *Col10a1 *in control bones (**B**; data represent means ± SD from three independent trials). Expression patterns of selected chondrocyte marker genes under control conditions in our microarray data sets further demonstrated efficient separation of regions (**C**).

We next performed microarray analyses to identify target genes of CNP in chondrocytes. Tibiae were cultured for six days in the absence or presence of CNP and then micro-dissected into three distinct zones: the resting/proliferative (RP), the hypertrophic (H), and the mineralized (M) zones (Fig. 5A). RNA was isolated directly from tibiae from three independent trials for each zone and both treatments were analyzed using Affymetrix Mouse 2.0 arrays in the London Regional Genomics Center as described (London, Ontario, Canada) [28]. Real-time PCR analyses of collagen II (*Col2a1*) and collagen X (*Col10a1*), known markers of cartilage development, confirmed that micro-dissection resulted in efficient separation of the zones (Fig. 5B). Microarray profiles of selected genes involved in endochondral bone growth are shown to further illustrate correct separation of zones (Fig. 5C).

**Figure 6:**
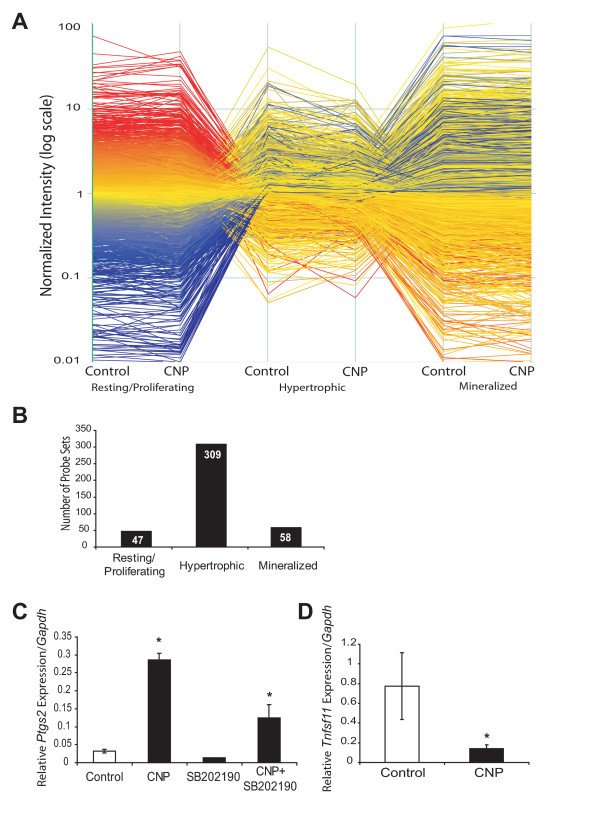
**Microarray analyses identify the hypertrophic area as the main target of CNP treatment**. E15.5 tibiae were isolated, incubated with or without CNP (1 μM) and DMSO or SB202190 (10 μM) and micro-dissected into the resting/proliferating, hypertrophic, and mineralized regions prior to RNA extraction and microarray analyses. Analyses of microarray results from three independent trials using Genespring 7.2 (**A**) illustrated that the hypertrophic zone was most significantly responsive to CNP treatment, when compared to control conditions (**B**). Six times as many probe sets showed at least 2-fold expression changes in the hypertrophic zone when compared to either resting/proliferating or mineralized regions. Real-time PCR analyses on micro-dissected tibiae were used to validate selected microarray patterns. CNP induction of *Ptgs2*, the gene encoding cyclooxygenase-2, was confirmed (**C**). SB202190 treatment did reduce basal Cox2 mRNA levels, but did not interfere with CNP induction of Cox2. *Tnfsf11*, the gene encoding RANKL, was confirmed to be down-regulated in response to CNP treatment. Data represent means ± SD of three independent trials (p < 0.05).

Bioinformatics analyses of microarray results (Fig. 6A) demonstrated that the hypertrophic zone was most responsive to CNP (Fig. 6B). Only 47 probe sets in the resting/proliferative zone (35 down, 12 up) and 58 probe sets in the mineralized zone (41 down, 17 up) responded with minimum two-fold responses to CNP (see Table 1 and 2 for lists of regulated genes). In contrast, 309 probe sets in the hypertrophic area showed a two-fold or higher change in expression in response to CNP. Of these probe sets, 157 probe sets were up-regulated by CNP in the hypertrophic zone, and 152 were down-regulated by CNP (Table 3).

**Table 1:** Genes showing 2-fold or greater changes in resting/proliferating zone

**Gene Name**	**Gene Description**	**Fold Change (CNP/Control)**
Spock3	sparc/osteonectin, cwcv and kazal-like domains proteoglycan 3	2.76
Il15	interleukin 15	2.73
Mgst2	microsomal glutathione S-transferase 2	2.60
Alcam	activated leukocyte cell adhesion molecule	2.52
Tec	cytoplasmic tyrosine kinase, Dscr28C related (Drosophila)	2.27
Aard	alanine and arginine rich domain containing protein	2.24
Tnfsf11	tumor necrosis factor (ligand) superfamily, member 11	2.18
Vegfc	vascular endothelial growth factor C	2.18
Senp8	SUMO/sentrin specific protease family member 8	2.09
Pde4b	phosphodiesterase 4B, cAMP specific	2.02
		
Gcnt2	glucosaminyl (N-acetyl) transferase 2, I-branching enzyme	0.18
Cidea	cell death-inducing DNA fragmentation factor, alpha subunit-like	0.24
Frzb	frizzled-related protein	0.28
F5	coagulation factor V	0.31
Car8	carbonic anhydrase 8	0.32
Ibsp	integrin binding sialoprotein	0.34
Lifr	leukemia inhibitory factor receptor	0.36
Col10a1	procollagen, type X, alpha 1	0.37
Lepr	leptin receptor	0.37
Ibsp	integrin binding sialoprotein	0.38
Pthr1	parathyroid hormone receptor 1	0.38
Clcn7	chloride channel 7	0.41
Chd7	chromodomain helicase DNA binding protein 7	0.42
H2-DMa	histocompatibility 2, class II, locus DMa	0.42
Col13a1	procollagen, type XIII, alpha 1	0.42
Cntn1	contactin 1	0.43
Slc7a3	solute carrier family 7, member 3	0.44
Tm7sf1	transmembrane 7 superfamily member 1	0.45
Iqsec1	IQ motif and Sec7 domain 1	0.45
Pthlh	parathyroid hormone-like peptide	0.46
Pdzk3	PDZ domain containing 2	0.46
Tmie	transmembrane inner ear	0.47
Ifitm5	interferon induced transmembrane protein 5	0.47
Socs2	suppressor of cytokine signaling 2	0.47
Angptl2	angiopoietin-like 2	0.48
Capn6	calpain 6	0.48
Atp10d	ATPase, Class V, type 10D	0.48
Cald1	caldesmon 1	0.49
Phtf2	putative homeodomain transcription factor 2	0.50

**Table 2:** Genes showing 2-fold or greater changes in mineralized zone

**Gene Name**	**Gene Description**	**Fold Change (CNP/Control)**
Ptgs2	prostaglandin-endoperoxide synthase 2	2.87
Bcan	brevican	2.75
Gabrb3	gamma-aminobutyric acid (GABA-A) receptor, subunit beta 3	2.46
Robo4	roundabout homolog 4 (Drosophila)	2.31
Cd38	CD38 antigen	2.23
Lepr	leptin receptor	2.22
Cd24a	CD24a antigen	2.15
Tgfbi	transforming growth factor, beta induced	2.14
Tmem56	transmembrane protein 56	2.12
Gsg2	germ cell-specific gene 2	2.11
Dspg3	dermatan sulphate proteoglycan 3	2.11
Siat4c	sialyltransferase 4C (beta-galactoside alpha-2,3-sialytransferase)	2.04
		
Hbb-y	hemoglobin Y, beta-like embryonic chain	0.21
Zbtb8	zinc finger and BTB domain containing 8	0.24
Chic1	cysteine-rich hydrophobic domain 1	0.27
Mia	EGL nine homolog 2 (C. elegans)	0.28
Nox4	NADPH oxidase 4	0.29
Fgd6	FYVE, RhoGEF and PH domain containing 6	0.32
Ddc	dopa decarboxylase	0.34
Crxos1	Crx opposite strand transcript 1	0.37
Hapln1	cartilage link protein 1	0.37
Col27a1	procollagen, type XXVII, alpha 1	0.38
Fgd5	FYVE, RhoGEF and PH domain containing 5	0.38
Msi2h	Musashi homolog 2 (Drosophila)	0.39
Rgs11	regulator of G-protein signaling 11	0.42
Mrpl35	mitochondrial ribosomal protein L35	0.43
Wwp2	WW domain containing E3 ubiquitin protein ligase 2	0.44
Glt25d2	glycosyltransferase 25 domain containing 2	0.44
Zcchc5	zinc finger, CCHC domain containing 5	0.45
Stno	strawberry notch homolog (Drosophila)	0.46
Ppp1r3c	protein phosphatase 1, regulatory (inhibitor) subunit 3C	0.46
Col9a3	procollagen, type IX, alpha 3	0.47
Igf2	insulin-like growth factor 2	0.47
Edil3	EGF-like repeats and discordin I-like domains 3	0.48
Ttll3	tubulin tyrosine ligase-like family, member 3	0.48
A2m	alpha-2-macroglobulin	0.49
Ctf1	cardiotrophin 1	0.49
Xist	inactive X specific transcripts	0.49
Zfp458	zinc finger protein 458	0.50

**Table 3:** Genes showing 2-fold or greater changes in hypertrophic zone

**Gene Name**	**Gene Description**	**Fold Change (CNP/Control)**
Cxcl14	chemokine (C-X-C motif) ligand 14	7.15
Ptgs2	prostaglandin-endoperoxide synthase 2	6.77
Grem1	cysteine knot superfamily 1, BMP antagonist 1	6.47
Fbxo32	F-box only protein 32	5.87
Glipr1	GLI pathogenesis-related 1 (glioma)	5.22
Gdf5	growth differentiation factor 5	4.93
Nox4	NADPH oxidase 4	3.78
Ebi2	Epstein-Barr virus induced gene 2	3.78
Evi1	ecotropic viral integration site 1	3.50
Prnd	prion protein dublet	3.43
Acdc	adipocyte complement related protein	3.42
Nes	nestin	3.33
Tnnt3	troponin T3, skeletal, fast	3.29
Nox4	NADPH oxidase 4	3.27
Rbp1	retinol binding protein 1, cellular	3.19
Hist1h2bc	histone 1, H2bp	3.16
Inhbb	inhibin beta-B	3.12
Sox17	SRY-box containing gene 17	3.10
Rnf125	ring finger protein 125	3.08
Fabp4	fatty acid binding protein 4, adipocyte	3.07
C1ql3	C1q-like 3	3.03
Rbpms	RNA binding protein gene with multiple splicing	3.02
Nrarp	Notch-regulated ankyrin repeat protein	3.02
Mmrn2	multimerin 2	3.00
Cldn5	claudin 5	3.00
Cd44	CD44 antigen	2.99
Klhl4	kelch-like 4 (Drosophila)	2.98
Pscd4	pleckstrin homology, Sec7 and coiled/coil domains 4	2.90
Ptprc	protein tyrosine phosphatase, receptor type, C	2.86
Rasgrp1	RAS guanyl releasing protein 1	2.85
Ptger2	prostaglandin E receptor 2 (subtype EP2)	2.84
Ctla2b	trophoblast specific protein beta	2.84
Copg2as2	coatomer protein complex, subunit gamma 2, antisense 2	2.82
Ian1	immune associated nucleotide 1	2.82
Pmaip1	phorbol-12-myristate-13-acetate-induced protein 1	2.77
Gpihbp1	GPI-anchored HDL-binding protein 1	2.73
Cdh5	cadherin 5	2.69
Niban	niban protein	2.67
Ptpn3	protein tyrosine phosphatase, non-receptor type 3	2.67
Slc26a7	solute carrier family 26, member 7	2.67
Tm6sf1	transmembrane 6 superfamily member 1	2.65
Pkp2	plakophilin 2	2.65
Bcl2a1a	B-cell leukemia/lymphoma 2 related protein A1a	2.63
Prss8	protease, serine, 8 (prostasin)	2.61
Fads3	fatty acid desaturase 3	2.60
Runx1	runt related transcription factor 1	2.56
Abcc9	ATP-binding cassette, sub-family C (CFTR/MRP), member 9	2.56
Nr2f1	nuclear receptor subfamily 2, group F, member 1	2.55
Hbb-y	hemoglobin Y, beta-like embryonic chain	2.52
Akr1b8	aldo-keto reductase family 1, member B8	2.51
Siat8f	sialyltransferase 8 (alpha-2, 8-sialyltransferase) F	2.51
Sfpi1	SFFV proviral integration 1	2.51
Zbtb33	zinc finger and BTB domain containing 33	2.49
Gdpd1	glycerophosphodiester phosphodiesterase domain containing 1	2.46
Clecsf6	C-type lectin, superfamily member 6	2.46
Pstpip1	proline-serine-threonine phosphatase-interacting protein 1	2.45
Esam1	endothelial cell-specific adhesion molecule	2.44
Cdh13	cadherin 13	2.43
Hist2h3c2	histone 2, H2aa1	2.43
Sfrp2	secreted frizzled-related sequence protein 2	2.40
Cables1	Cdk5 and Abl enzyme substrate 1	2.39
Ednrb	endothelin receptor type B	2.39
Eltd1	EGF, latrophilin seven transmembrane domain containing 1	2.38
Calcrl	calcitonin receptor-like	2.38
Ctla2b	trophoblast specific protein beta	2.38
Ian1	immune associated nucleotide 1	2.36
Sox18	SRY-box containing gene 18	2.36
Plce1	phospholipase C, epsilon 1	2.33
Il13ra1	interleukin 13 receptor, alpha 1	2.33
Cd38	CD38 antigen	2.32
Ncf4	neutrophil cytosolic factor 4	2.30
Rgs4	regulator of G-protein signaling 4	2.30
Ptpn8	protein tyrosine phosphatase, non-receptor type 8	2.29
Inhba	inhibin beta-A	2.29
Alcam	activated leukocyte cell adhesion molecule	2.27
Pira1	paired-Ig-like receptor A1	2.27
Cav2	caveolin 2	2.27
Cxcr4	chemokine (C-X-C motif) receptor 4	2.26
Sh3bp5	calpain 7	2.26
Mfap3l	microfibrillar-associated protein 3-like	2.26
Dscr1	Down syndrome critical region homolog 1 (human)	2.25
Mcam	melanoma cell adhesion molecule	2.24
Ms4a6d	membrane-spanning 4-domains, subfamily A, member 6D	2.24
Cd34	CD34 antigen	2.24
Zfp42	zinc finger protein 42	2.23
Kcne3	potassium voltage-gated channel, Isk-related subfamily, gene 3	2.22
Ivns1abp	influenza virus NS1A binding protein	2.22
Cd84	CD84 antigen	2.22
Kdr	kinase insert domain protein receptor	2.21
Clca5	chloride channel calcium activated 5	2.20
Itga9	integrin alpha 9	2.19
Prkch	protein kinase C, eta	2.19
Tex15	testis expressed gene 15	2.18
Plac8	placenta-specific 8	2.17
Ebf3	early B-cell factor 3	2.16
Lcp2	lymphocyte cytosolic protein 2	2.16
Mcoln3	mucolipin 3	2.15
Sh3glb1	SH3-domain GRB2-like B1 (endophilin)	2.15
Ugt1a2	UDP glycosyltransferase 1 family, polypeptide A6	2.15
Egfl7	EGF-like domain 7	2.15
Icam2	intercellular adhesion molecule 2	2.15
Six1	sine oculis-related homeobox 1 homolog (Drosophila)	2.14
Chst7	carbohydrate (N-acetylglucosamino) sulfotransferase 7	2.13
Evi2a	ecotropic viral integration site 2a	2.12
Myct1	myc target 1	2.12
Pde4b	phosphodiesterase 4B, cAMP specific	2.12
Adamts1	a disintegrin-like & metalloprotease with thrombospondin type 1	2.10
Snx10	sorting nexin 10	2.10
Rac2	RAS-related C3 botulinum substrate 2	2.09
Siat8d	sialyltransferase 8 (alpha-2, 8-sialyltransferase) D	2.08
Dsg2	desmoglein 2	2.07
F11r	F11 receptor	2.06
Lrrc33	leucine rich repeat containing 33	2.06
Ian9	Similar to hypothetical protein (LOC243374), mRNA	2.06
Slc30a1	solute carrier family 30 (zinc transporter), member 1	2.05
Kcnj8	potassium inwardly-rectifying channel, subfamily J, member 8	2.05
Cotl1	coactosin-like 1 (Dictyostelium)	2.04
Ptx3	pentaxin related gene	2.04
Ctla2b	trophoblast specific protein beta	2.03
Sipa1	signal-induced proliferation associated gene 1	2.03
Rgs5	regulator of G-protein signaling 5	2.03
Itgax	integrin alpha X	2.01
Car2	carbonic anhydrase 2	2.01
Serpind1	serine (or cysteine) proteinase inhibitor, clade D, member 1	2.01
Cadps2	Ca2+-dependent activator protein for secretion 2	2.00
Il1rl2	interleukin 1 receptor-like 2	2.00
		
Lemd1	LEM domain containing 1	0.06
Gzme	granzyme E	0.14
Plekha7	pleckstrin homology domain containing, family A member 7	0.14
Chad	chondroadherin	0.15
Cd28	CD28 antigen	0.17
Sep-04	septin 4	0.19
Pltp	phospholipid transfer protein	0.19
Il15	interleukin 15	0.20
Ttll3	tubulin tyrosine ligase-like family, member 3	0.20
Syt8	synaptotagmin 8	0.20
Gpr91	G protein-coupled receptor 91	0.23
Sep-04	septin 4	0.24
Cd28	CD28 antigen	0.24
Efemp1	epidermal growth factor-containing fibulin-like ECM protein 1	0.25
Tnfsf11	tumor necrosis factor (ligand) superfamily, member 11	0.27
Tlr1	toll-like receptor 1	0.28
Trim2	tripartite motif protein 2	0.28
Vnn1	vanin 1	0.28
Tnni2	troponin I, skeletal, fast 2	0.29
Enpp6	ectonucleotide pyrophosphatase/phosphodiesterase 6	0.30
Fxyd2	FXYD domain-containing ion transport regulator 2	0.30
Rtn2	reticulon 2 (Z-band associated protein)	0.31
Iqgap2	IQ motif containing GTPase activating protein 2	0.31
Capn6	calpain 6	0.32
Rab27a	RAB27A, member RAS oncogene family	0.32
Aicda	activation-induced cytidine deaminase	0.33
F5	coagulation factor V	0.33
Hs6st2	heparan sulfate 6-O-sulfotransferase 2	0.33
Cklfsf8	chemokine-like factor super family 8	0.33
Nrk	Nik related kinase	0.33
Gprasp2	G protein-coupled receptor associated sorting protein 2	0.33
Car8	carbonic anhydrase 8	0.34
Prom1	prominin 1	0.34
Mgst2	microsomal glutathione S-transferase 2	0.34
Pltp	phospholipid transfer protein	0.35
Stc2	stanniocalcin 2	0.35
Lipg	lipase, endothelial	0.35
Il17d	interleukin 17D	0.36
Serpinb6b	serine (or cysteine) proteinase inhibitor, clade B, member 6b	0.36
Matn3	matrilin 3	0.37
Slc1a1	solute carrier family 1, member 1	0.37
Art3	ADP-ribosyltransferase 3	0.37
Cp	ceruloplasmin	0.37
Abi3bp	ABI gene family, member 3 (NESH) binding protein	0.37
Matn1	matrilin 1, cartilage matrix protein 1	0.38
A2m	alpha-2-macroglobulin	0.38
Usp11	ubiquitin specific protease 11	0.38
Col9a2	procollagen, type IX, alpha 2	0.39
Pik3r1	phosphatidylinositol 3-kinase, regulatory subunit, polypeptide 1	0.39
Rlbp1	retinaldehyde binding protein 1	0.39
Rnase4	ribonuclease, RNase A family 4	0.39
Col14a1	procollagen, type XIV, alpha 1	0.40
Slc19a3	solute carrier family 19 (sodium/hydrogen exchanger), member 3	0.40
Nfkbiz	NFK light polypeptide gene enhancer in B-cells inhibitor, zeta	0.40
Slco2b1	solute carrier organic anion transporter family, member 2b1	0.41
Fxyd6	FXYD domain-containing ion transport regulator 6	0.41
Egr3	early growth response 3	0.41
Usp53	ubiquitin specific peptidase 53	0.41
Serpini1	serine (or cysteine) proteinase inhibitor, clade I, member 1	0.41
Pitpnc1	phosphatidylinositol transfer protein, cytoplasmic 1	0.42
Anxa8	annexin A8	0.42
Il17b	interleukin 17B	0.43
Gpr126	G protein-coupled receptor 126	0.43
Pank1	pantothenate kinase 1	0.43
Dock9	dedicator of cytokinesis 9	0.43
Sfmbt2	Scm-like with four mbt domains 2	0.43
Enpp2	ectonucleotide pyrophosphatase/phosphodiesterase 2	0.44
Kctd4	potassium channel tetramerisation domain containing 4	0.44
Cobll1	Cobl-like 1	0.44
Scrg1	scrapie responsive gene 1	0.45
Matn3	matrilin 3	0.45
Zfpm2	zinc finger protein, multitype 2	0.45
Lims2	LIM and senescent cell antigen like domains 2	0.45
Gpr64	G protein-coupled receptor 64	0.45
Ptprz1	protein tyrosine phosphatase, receptor type Z, polypeptide 1	0.46
Hhip	Hedgehog-interacting protein	0.46
Eps8	epidermal growth factor receptor pathway substrate 8	0.46
Heph	hephaestin	0.47
Sesn1	sestrin 1	0.47
Ctf1	cardiotrophin 1	0.47
Zfp612	zinc finger protein 612	0.48
Wdr40b	WD repeat domain 40B	0.48
Dkk1	dickkopf homolog 1 (Xenopus laevis)	0.48
Ogt	O-linked N-acetylglucosamine (GlcNAc) transferase	0.48
Ddc	dopa decarboxylase	0.48
Adam17	a disintegrin and metallopeptidase domain 17	0.48
Rdhe2	short chain dehydrogenase reductase 9	0.48
Vav3	vav 3 oncogene	0.48
Tmem56	transmembrane protein 56	0.48
Aldh1a3	aldehyde dehydrogenase family 1, subfamily A3	0.48
Zfp521	ecotropic viral integration site 3	0.48
Fbxo25	F-box only protein 25	0.49
Kitl	kit ligand	0.49
Plagl1	pleiomorphic adenoma gene-like 1	0.49
Hectd2	HECT domain containing 2	0.49
Bmper	BMP-binding endothelial regulator	0.49
Gprasp1	G protein-coupled receptor associated sorting protein 1	0.50
Gdf10	growth differentiation factor 10	0.50
Sox5	SRY-box containing gene 5	0.50

One of the genes showing a strong increase in the hypertrophic zone (>6-fold) was *Ptgs2*, encoding cyclooxygenase 2 (Cox2), a key enzyme in the synthesis of prostaglandins. Since *Ptgs2 *and its products (such as prostaglandin E2) are known to play important roles in chondrocyte differentiation and skeletal remodeling [29-31], we selected this gene for validation experiments. Induction of Cox2 mRNA expression in the hypertrophic zone by CNP was confirmed by real-time PCR, which showed a 10-fold increase in transcript levels (Fig. 6C). p38 inhibition did reduce the basal levels of Cox2 mRNA, but surprisingly did not affect the induction by CNP. Among the genes down-regulated by CNP was the *Tnfsf11 *gene, encoding RANKL, a known activator of osteoclastic resorption of bone and cartilage [32,33]. *Tnfsf11 *displayed a 3.8-fold reduction in expression according to microarray analyses. Because down-regulation of *Tnfsf11 *could provide a molecular mechanism for the observed delay in mineralization and cartilage remodeling in response to CNP, we chose to validate its expression. Real-time PCR analysis confirmed down-regulation of *Tnfsf11 *mRNA levels in the hypertrophic zone by CNP (Fig 6D).

**Figure 7:**
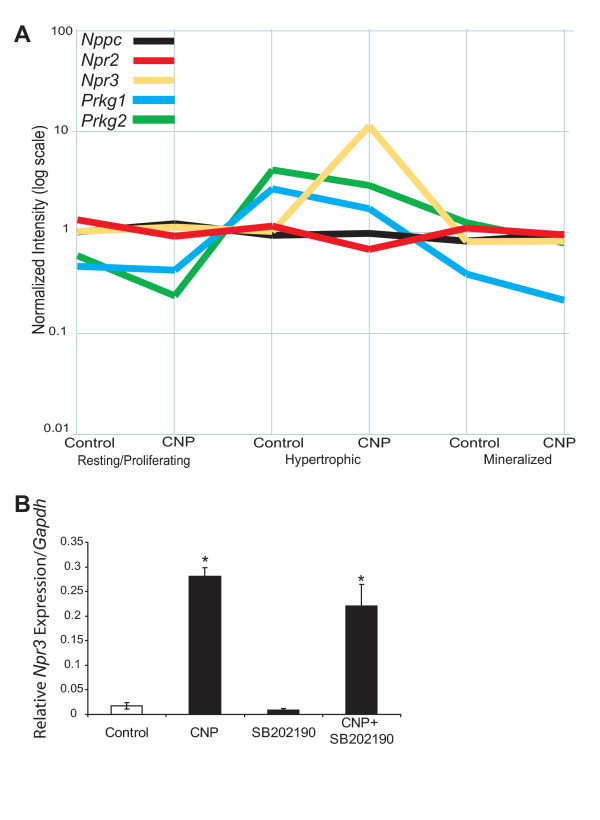
**Expression patterns from microarray analyses demonstrate up-regulation of cGMP-dependent kinase genes in the hypertrophic zone**. Microarray analyses of the principal players in the CNP pathway in micro-dissected tibiae cultured with and without CNP (1 μM) are shown (**A**). *Prkg1 *and *Prgk2*, encoding cGMP-dependent kinases I and II, were strongly up-regulated in the hypertrophic zone, irrespectively of exogenous CNP. In addition, CNP strongly stimulated expression of *Npr3*, the natriuretic peptide clearance receptor, in the hypertrophic zone. Real-time analysis confirmed induction of *Npr3 *by CNP, which primarily occurs through a p38-independent manner. Data represent means ± SD of three independent trials (p < 0.05).

To answer the question why the hypertrophic zone is much more responsive to CNP treatment than other zones, we examined expression of key genes in the CNP signaling pathway. Analyses of our microarray data demonstrated that the genes encoding CNP (*Nppc*), its signaling receptor GC-B (*Npr2*) and the decoy receptor (*Npr3*) are expressed at similar levels in all three zones of micro-dissected tibiae under control conditions (Fig. 7A). However, *Prkg1 *(encoding cGMP-dependent kinase I) expression is 5.9 fold higher in hypertrophic chondrocytes than in the resting/proliferative cells, and seven-fold higher in the hypertrophic versus the mineralized zone (Fig. 7A). Similarly, *Prkg2 *expression is 4.4-fold and 2.5-fold higher in the hypertrophic zone versus the resting/proliferative and mineralized zones, respectively (Fig. 7A). This expression pattern of key mediators of CNP signaling can explain the strong responsiveness of hypertrophic chondrocytes to CNP. In addition, our microarray data on expression of the decoy receptor *Npr3 *in the hypertrophic zone, while variable and thus not statistically significant, suggested that CNP strongly activates the expression of *Npr3*. We therefore decided to analyze its expression by real-time PCR which demonstrated a statistically significant 16-fold induction of *Npr3 *expression in the hypertrophic zone by CNP that was not altered by p38 inhibition (Fig. 7B). CNP did not affect *Npr3 *expression in the other growth plate zones.

### Annotation of microarray data identifies CNP-regulated pathways

**Figure 8:**
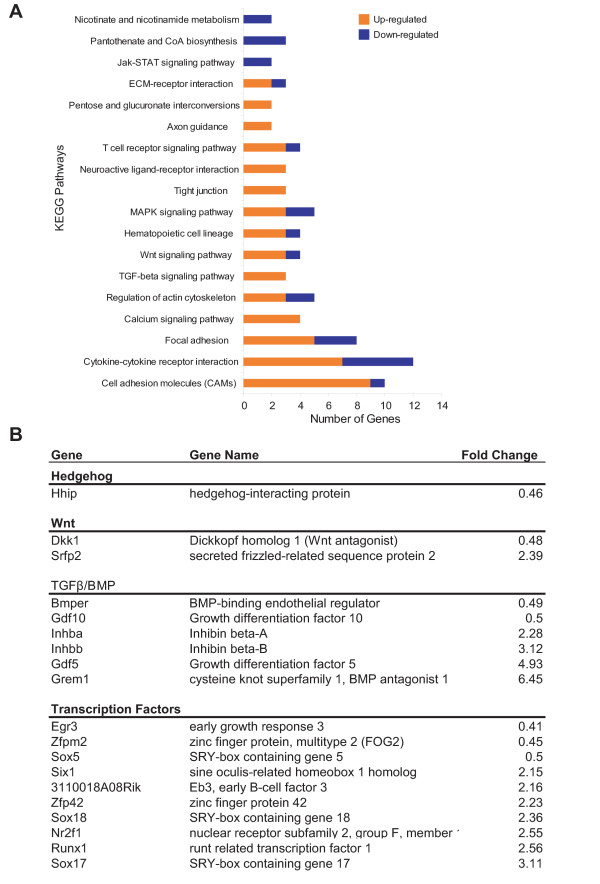
**Detailed analyses of microarray data identify CNP-regulated pathways**. Microarray data sets from hypertrophic areas of micro-dissected tibiae cultured with and without CNP (1 μM) were analyzed using KEGG annotations (**A**). Genes up- and down-regulated by CNP contributed approximately proportionally to many pathways. However, up-regulated genes dominated the cell adhesion molecules, TGFbeta and calcium signaling and tight junction categories (among others). Fold change of selected genes in the BMP/GDF, Wnt and hedgehog pathways in response to CNP is shown (as ratio of CNP to control; **B**). A list of transcription factor genes regulated by CNP is also shown.

To gain insight into biological processes regulated by CNP, we employed KEGG annotation [34] on genes showing at least two-fold changes in response to CNP in the hypertrophic area (Fig. 8A). Numerous pathways were affected by CNP, most of them comprised approximately proportionally by up- and down-regulated genes. However, genes related to cell adhesion were strongly enriched in up-regulated genes, suggesting that CNP promotes cell adhesion. Most notably, CNP induced expression of genes involved in cell-cell interactions such as *Icam2 *(intercellular adhesion molecule 2), *Cdh5 *(Cadherin 5), and *Esam1 *(endothelial cell-specific adhesion molecule). In contrast, down-regulated genes included many genes encoding extracellular matrix molecules, for example *Matn1 *and *Matn3 *(Matrilin 1 and 3), *Col9a2 *(procollagen type IX, alpha 2) and *Col14a1 *(procollagen type XIV, alpha 1) (Table 3). In addition, up-regulated genes included three members of the TGFβ superfamily, *Gdf5, Inhbb *and *Inhba*, as well as the BMP antagonist *Grem1 *(Fig. 8B). Besides the TGFβ family, members of the Wnt and hedgehog signaling pathways are important regulators of cartilage differentiation, and components of these pathways were regulated by CNP. Other categories in which up-regulated genes were over-represented included tight junctions and calcium signaling, whereas pantothenate and CoA biosynthesis was one example for a pathway dominated by down-regulated genes. Finally, more transcription factor-encoding genes were up-regulated than down-regulated by CNP (Fig. 8B).

We also performed KEGG analyses of CNP-regulated genes in the other zones. In the proliferative zone, genes involved in cytokine receptor interactions were most prominent with four genes, three of each were upregulated by CNP (Table 1). The categories of cell adhesion and focal adhesion molecules were represented by three genes each (data not shown). In contrast, no category was represented by more than two genes in the mineralized zone (data not shown).

## Discussion

Gene disruption and other studies have identified the CNP pathway as one of the most important anabolic regulators of endochondral bone growth. However, the molecular and cellular mechanisms involved are not completely understood. Here we provide multiple novel insights into these mechanisms. Most importantly, we show that the p38 MAP kinase pathway is an essential mediator of CNP effects on endochondral bone growth. Second, we identify the hypertrophic zone of the growth plate as the main target of CNP signaling, likely because of the high levels of cGMP-dependent kinase I and II expression in this zone. Third, we used genome-wide microarray analyses to identify multiple target genes potentially involved in CNP effects in cartilage.

Earlier studies have demonstrated that CNP stimulates bone growth through enhanced proliferation, mineralization and extracellular matrix synthesis [10,12,13]. Our data suggest that effects of CNP on longitudinal bone growth are largely due to the expansion of the hypertrophic zone, in agreement with earlier studies [23]. This could be due, in principle, to a number of effects, such as increased rate of generation of hypertrophic chondrocytes, increased size of individual hypertrophic chondrocytes, and delayed replacement of hypertrophic cartilage by bone. It should be noted that these possibilities are not exclusive, and several or all of them can contribute to the observed effects of CNP. Multiple observations support the notion that delayed removal of hypertrophic chondrocytes is one of the mechanisms involved in CNP-induced bone growth. First, terminal hypertrophic chondrocytes at the metaphysis reach a larger size, suggesting that a delay in chondrocyte replacement by bone tissue allows for a longer period of cellular growth. Furthermore, while CNP increases the size of the entire tibia significantly, this increase is not matched by a proportional increase in the area of the mineralized region. This observation suggests that CNP delays remodeling of the metaphysis and the replacement of cartilage by bone. Finally, our microarray data show that expression of RANKL, a potent activator of osteoclastic bone resorption, in the hypertrophic zone is down-regulated by CNP. RANKL is expressed in hypertrophic cartilage [35-37], where it likely stimulates the removal of hypertrophic cartilage by osteoclasts and facilitates vascular invasion and ossification. Repression of RANKL expression by CNP could thus delay these remodeling events. Experiments are under way in our laboratory to examine whether osteoclast activity is indeed reduced in CNP-treated organ cultures.

We and others have shown important roles of p38 in hypertrophic chondrocyte differentiation *in vitro *and *in vivo *[3,27,38-41]. Thus, it is not surprising that p38 inhibition reverses CNP effects on longitudinal growth and the expansion of the hypertrophic zone. Moreover, our data show that p38 activity is required for the repression of mineralization by CNP. These data are in agreement with a recent study showing delayed primary and secondary ossification in transgenic mice overexpressing an activated form of MKK6, an upstream activator of p38, in cartilage [27]. It should be noted, however, that other phenotypes of these mice (such as reduced proliferation and delayed hypertrophy) are not recapitulated in our studies, potentially due to altered patterns and/or levels of p38 activation in the two studies (e.g. transgenic expression of activated MKK6 under the collagen II promoter versus activation of p38 through the endogenous NPR2/cGMP signaling cascade) or because CNP acts through additional pathways besides p38. Independent of these complications, our studies provide strong evidence for a novel function of p38 signaling in maintaining hypertrophic cartilage and delaying the replacement of cartilage by bone.

However, our data also show that p38 signaling is not required for all effects of CNP on hypertrophic cartilage. While p38 inhibition results in lower basal levels of Cox2 mRNA in chondrocytes, in agreement with observations by other studies [42-44], CNP still causes a strong increase in Cox2 expression in the presence of SB202190. Similarly, *Npr3 *induction by CNP is independent of p38 activity. Therefore, it appears likely that p38 signaling is required to achieve and/or maintain the expanded hypertrophic zone in CNP-treated bones, but not for induction of some target genes. Studies to identify additional signaling pathways connecting CNP to Cox2 gene expression are underway in our laboratory.

Our studies also demonstrate antagonistic roles of p38 and another MAP kinase pathway, the MEK1/2-ERK1/2 pathway. Inhibition of MEK1/2 activity results in enhanced growth of endochondral bones, with no additive or synergistic effect with CNP. While our studies were in progress, other groups showed that the MEK1/2-ERK1/2 indeed reduces endochondral bone growth *in vivo *[26] and that CNP inhibits ERK1/2 activity [21], in agreement with our studies. Additional studies confirmed the close and reciprocal interactions between CNP-cGMP and FGF-MEK1/2-ERK signaling [22,23]. For example, CNP was shown to repress FGF-induced growth arrest and extracellular matrix degradation by counteracting MEK1/2 activation, while FGFs 2 and 18 suppress CNP-stimulated cGMP production [22,23]. However, none of these studies evaluated a potential role of p38 signaling in this context. Since p38 has also been implicated in FGF signal transduction in chondrocytes [45,46], it will be interesting to investigate whether this MAP kinase is involved in the antagonistic effects of CNP and FGF in endochondral bone growth. However, both CNP and FGFs activate p38 in chondrocytes, but they have opposing effects on the growth of endochondral bones. The role of p38 in FGF effects on chondrocytes has, to our knowledge, only been studied in cell culture, not in a three-dimensional model that allows direct assessment of bone growth. Based on our data, we don't expect that p38 activation contributes to the growth-repressing activities of FGF, but this prediction needs to be experimentally verified. Nevertheless, the fact that both FGFs and CNP activate p38 despite their opposing effects on bone growth makes it unlikely that p38 contributes to crosstalk between the two signaling systems, in contrast to ERK1/2.

Similarly, it will be important to examine whether regulation of the different MAP kinases by CNP occurs through independent, parallel pathways, or whether they regulate each other. In addition, the pathways connecting CNP to the MAP kinase modules have not been completely resolved. For example, while it has been shown that repression of ERK activity by CNP occurs at the level of the upstream kinase Raf1 and requires cGMP-dependent kinase activity [22], the exact molecular mechanism involved has not been described.

This is, to our knowledge, one of the first studies to use micro-dissection of mammalian endochondral bones for genome-wide expression analyses by microarrays. We chose to perform these studies after 6 days of CNP stimulation, as opposed to a short term treatment. While this approach does not allow us to distinguish direct and indirect target genes of CNP, it mimics the *in vivo *situation where cells are exposed to auto-/paracrine CNP signaling for extended periods. Our study should therefore identify genes that are regulated by long term exposure to CNP and are thus likely to be involved in the physiological activities of CNP in the growing skeleton. Our microarray data were confirmed by real-time PCR analyses for selected genes; these data and our earlier studies [28,47,48] strongly suggest that the vast majority of the expression profiles detected by our microarrays correspond to the authentic gene expression patterns. Analyses of array data as well as confirmatory real-time PCRs (e.g. for type X collagen) also demonstrated that our micro-dissection protocol results in efficient separation of different zones of the cartilage and can be used for identification of novel hypertrophy-specific genes. Moreover, these data clearly show that the hypertrophic zone of the growth plate is by far the most responsive to CNP. This responsiveness does not correlate to altered levels of mRNAs for CNP itself, its signaling receptor, or the decoy receptor in control conditions. Instead, our data suggest that the expression of cGMP-dependent kinases I and II (cGKI, II) is much higher in this zone than in the other ones, providing further evidence for a crucial role of these enzymes in CNP signal transduction. Interestingly, the expression of these two genes, as well as the *Nppc *and *Npr2 *genes, in the hypertrophic zone is not altered in response to CNP. In contrast, *Npr3 *expression is strongly induced by CNP in the hypertrophic zone. While this induction was not identified as significant in the microarray analyses, subsequent real-time PCR confirmed the existence of this previously unknown feedback loop that likely limits CNP effects in growing cartilage.

Our expression data suggest that both cGKI and II are involved in mediating CNP effects on cartilage development. Studies with genetically altered mice and naturally occuring rat mutants demonstrate that cGKII is the dominating protein in chondrocytes [19,49]; however, the cartilage phenotypes of cGKII- and CNP-deficient mice are not identical, suggesting the possibility of an additional role of cGKI in CNP signaling. Double knockout mice for both cGKI and II will be required to resolve this issue.

Thus, our data in conjunction with published studies support a model where basal CNP signaling promotes proliferation and extracellular matrix synthesis in growth plate chondrocytes. Once cells start to differentiate, they increase their expression of cGMP-dependent kinases and their responsiveness to CNP. This results in an extension of hypertrophic chondrocyte life and a delay in osteoclast and potentially vascular invasion, thus promoting maximal growth of hypertrophic chondrocytes and endochondral bone growth. At the same time, high levels of CNP signaling induce expression of *Npr3 *that ultimately limits CNP effects, allowing for expression of RANKL and for remodeling of the metaphysis. Experiments are under way to examine whether this model accurately describes cellular mechanisms of CNP signaling in endochondral ossification and to identify the molecular mechanisms involved.

Detailed analyses of our microarray data provided novel insights into biological processes regulated by CNP. CNP treatment induced the expression of several genes for cell-cell interactions in the hypertrophic area (as well as the resting/proliferating zone), while at the same time repressing genes for ECM proteins. Another process regulated by CNP is signaling by TGFβ family members. Most interestingly, CNP induces expression of *Gdf5 *and *Grem1*, both of which have been implicated in skeletal development. Loss-of-function mutations of *Gdf5 *have been identified as cause of reduced skeletal growth in human chondrodysplasias and brachypodism mice [50]. Interestingly, GDF5 has been shown to stimulate cell adhesion in chondrocytes [51], in agreement with our data showing increased expression of both *Gdf5 *and cell adhesion molecules in response to CNP. Therefore, GDF5 is an excellent candidate for mediating the anabolic effects of CNP. In contrast, *Grem1 *encodes a BMP antagonist that is required for limb development and controls chondrogenesis [52-56]. Moreover, *Grem1 *expression is induced by BMP/GDF signaling [57-59], suggesting that its stimulation could be secondary to increased expression of *Gdf5 *and/or related factors (e.g. *Inhbb *and *Inhba*) in response to CNP.

In summary, our results identify several novel components and characteristics of CNP signaling during endochondral bone growth. Collectively, these studies lead to the novel concept that CNP acts, at least in part, by delaying the terminal steps of endochondral ossification, i.e. the replacement of hypertrophic cartilage by bone. Further tests of this model *in vivo *and elucidation of the mechanisms involved will not only result in improved understanding of endochondral bone development, but will also be crucial for the development of potential therapeutic applications.

## Methods

### Materials

Timed-pregnant CD1 mice were purchased from Charles River Canada. CNP, 8-(4-cpt) cGMP, and pharmacological inhibitors were obtained from Sigma and Calbiochem. Cell culture reagents were from Invitrogen and general chemicals from VWR. All real-time PCR probes and reagents were purchased from Applied Biosystems. The phospho-MKK3/6 (Cat. number 9231) and phospho-p38 (9216) antibodies were from Cell Signaling Technologies, and the β-actin antibody was from Sigma.

### Organ Culture

Tibiae were isolated from embryonic day 15.5 (E15.5) embryos from CD1 timed-pregnant mice (Charles River Canada) using the Stemi DV4 Stereomicroscope (Zeiss). Dissection day was considered to be day 0 and tibiae were allowed to recover from dissection overnight in serum-free α-MEM media containing 0.2% Bovine Serum Albumin (BSA), 0.5 mM L-glutamine, 40 units penicillin/mL and 40 μg streptomycin/mL as described [60]. The following morning, bones in 24-well Falcon plates were measured using an eyepiece in the Stemi DV4 Stereomicroscope and treated with CNP (0.01 to 1 μM) or BSA/HCl (1 mM) vehicle, and DMSO or U0126, PD98059, PD169316, SB202190 or SB202474 (10 μM each). Media was changed every 48 hrs beginning on day 1, and bones measured on days 1, 3, 6, and 8. Results are expressed as change in length relative to day 1. Experiments were repeated at least three times, with 4–6 bones per treatment for each trial.

For weight determination and Alizarin Red/Alcian Blue staining, 6 bones per treatment were weighed at day 6 of culture and then placed in 4% Paraformaldehyde (PFA) in DEPC-treated PBS for overnight fixation. Subsequently, tibiae were placed in staining solution for 45–60 minutes (0.05% Alizarin Red, 0.015% Alcian Blue, 5% acetic acid in 70% ethanol). Images of stained bones were taken using a Nikon SMZ1500 dissecting microscope with Photometric CoolSNAP colour digital camera (Nikon Canada) and PTI Image Master 5 program. Stained areas in images were measured using Openlab 4.0.4 software program.

For experiments requiring perichondrium removal, tibiae were isolated from embryonic 15.5 day embryos under three different modes: very loose dissection ensuring that perichondrium was intact, very careful dissection in which perichondrium was removed mechanically, and treatment of tibiae with dispase (3 mg/mL in PBS) for 3–5 minutes with concurrent mechanical removal of perichondrium [61,62]. Media was changed every two days beginning on day 1 and bone lengths measured on days 1 and 6, with change in length expressed relative to day 1.

### Histology and Immunohistochemistry

After experiment completion, tibiae were rinsed with PBS and fixed in 4% PFA overnight. Bones were then stained with mercurochrome for visualization, placed in 10% formalin solution, and sent for embedding and sectioning in the Pathology lab at University of Western Ontario Hospital or the Molecular Pathology Core Facility at the Robarts Research Institute (London, Ontario, Canada). Following sectioning, bones were stained with hematoxylin and eosin using standard protocols. For immunohistochemistry, sections were incubated with primary anti-phospho-p38 antibody (1:50 dilution) over night at 4°C. Bound antibody was visualized using the UltraVision LPValue detection system (Lab Vision) with AEC chromogen substrate (Lab Vision).

### RNA isolation from organ cultures and microarray analyses

For experiments requiring RNA isolation from organ cultures, E15.5 tibiae were harvested and treated as described above with or without CNP and SB202190. On day 6 of treatment, tibiae were separated under a dissecting microscope into the resting/proliferative, hypertrophic, and mineralized areas. Same areas from approximately 24 bones were pooled per trial, in each of three independent trials. RNA was isolated following the RNeasy ^® ^Lipid Tissue Extraction protocol from Qiagen (Mississauga) and RNA integrity verified using the Agilent 2100 Bioanalyzer. Microarray analyses from three trials were performed at the London Regional Genomics Centre (London, Ontario, Canada) using MOE430 2.0 Affymetrix arrays consisting of 45,000 probe sets (covering the entire mouse genome). Results were analyzed using GeneSpring 7.2 software as described [28]. Microarray data were independently filtered using GeneSpring Bioscripts quality filters for noise and one-way ANOVA testing, to eliminate genes that were not expressed or showed great variability between replicates. The remaining 5199 probes sets common to both filtering methods were used for all subsequent analyses. Lists of genes undergoing at least two-fold changes were analyzed using the Babelomics suite [63] and in particular the KEGG pathways module in the FatigoPlus tool.

### Real-Time PCR

Real-Time PCR analysis was performed as described using Applied Biosystems 7900 HT Real-Time PCR System and TaqMan^® ^Gene Expression Assays [28,40,64]. All probes (*Npr3*, *Col2a1*, *Col10a1*, *Ptgs2*, *Tnfsf11 *and *Gapdh*)were purchased from Applied Biosystems. Gene expression levels were determined using the Standard Curve quantitative method with *Gapdh *levels as the basis of comparison.

### Statistical Analyses

All experiments were performed in at least three independent trials. Two-Way ANOVA (parametric) test with Bonferroni post-test were performed using the Graph Pad/Prism software. One-way ANOVA with Bonferroni post-test and paired t-tests were used when appropriate.

## List of abbreviations

BMP – bone morphogenetic protein

cGMP – cyclic guanosinemonophosphate

cGK – cGMP-dependent kinase

CNP – C-type natriuretic peptide

Cox2 – cyclooxygenase 2

GDF – growth differentiation factor

ERK – extracellular signal-regulated kinase

MEK – MAP/ERK kinase

PCR – polymerase chain reaction

RANKL – receptor activator of nuclear factor kappa B ligand

TGF – transforming growth factor

## Authors' contributions

H.A., S.K., R.G. and L-A.S. performed organ cultures and their analyses. H.A. and C.G.J. performed microarray analyses. R.S. provided consultation and training with organ cultures. F.B. conceived and designed the study and co-wrote the manuscript with H.A. All authors read and approved the final manuscript.
